# Obstructive Sleep Apnea with Paradoxical Vocal Cord Movement in Children during Sleep Endoscopy: Case Series

**DOI:** 10.1155/2020/7380802

**Published:** 2020-07-27

**Authors:** Maxwell Newby, Sarah Callaham, Michele Carr

**Affiliations:** ^1^Department of Otolaryngology, West Virginia University, Morgantown, USA; ^2^West Virginia University School of Medicine, Morgantown, USA; ^3^Department of Otolaryngology, Jacobs School of Medicine and Biomedical Sciences at the University at Buffalo, New York, USA

## Abstract

**Objective:**

To describe a series of pediatric cases of obstructive sleep apnea (OSA) with paradoxical vocal cord movement noted on drug-induced sleep endoscopy (DISE).

**Materials and Methods:**

Case series and chart review of children who had undergone DISE for OSA that showed PVCM.

**Results:**

Three cases where paradoxical vocal cord motion (PVCM) was noted during DISE are described. Two had an enlarged adenoid, and one had no other site of obstruction. Two were treated with adenoidectomy and antireflux medications. One received proton pump inhibitors alone. In all 3 cases, OSA symptoms resolved.

**Conclusion:**

This case series of documented obstructive sleep apnea related to paradoxical vocal cord movement suggests that this entity occurs during sleep with airway obstruction. Further clarification of etiology of PVCM during OSA and its management is needed.

## 1. Introduction

Paradoxical vocal cord movement (PVCM) was first described by Sir Mackenzie in 1869 as episodic paradoxical adduction of the true vocal cords, primarily during inspiration [1]. PVCM symptoms are frequently mistaken for asthma or other respiratory pathologies; however, its noted role as a potential cause of obstructive sleep apnea (OSA) has not been well characterized in the literature thus far. This case series presents three pediatric patients diagnosed with OSA by polysomnogram without known CNS disease and PVCM diagnosed during a drug-induced sleep endoscopy (DISE) with propofol infusion.

## 2. Case Series

This study was approved by the local Institutional Review Board. There are three pediatric patients presenting with OSA symptomatology who underwent DISE with propofol infusion revealing PVCM as a site of obstruction. The DISE procedure was modeled after Kezirian and performed in the same manner for all three cases [2]. The patients were not given preoperative sedation, anxiolytics, or topical medications. Oximetry and cardiac rhythm monitoring was performed throughout by the anesthesia team. The patients were placed supine on the operating room table and mask-ventilated with inhalant anesthesia to facilitate intravenous line placement. Intravenous propofol was started at 50–75 mcg/kg/min. The inhaled anesthesia was stopped, and the airway supported with blow-by oxygen by mask. The propofol dose was titrated to spontaneous breathing with palpable or audible upper airway air turbulence. At this point, a 2.7 mm flexible fiberoptic laryngoscope was passed through the nasal cavity into the upper airway and findings recorded. The evaluations were performed by the same pediatric otolaryngologist along with a resident, and in Case 3 with a pediatric pulmonologist as well.

### 2.1. Case 1

A 9-year-old female who was status post adenotonsillectomy with persistent sleep-disordered breathing symptoms including snoring, restless sleeping, frequent wakening, hyperactivity during the day, poor school performance, and daytime fatigue underwent a postoperative polysomnogram. This showed moderate OSA with an AHI of 5.4 and oxygen saturation nadir of 86%. Physical examination of the head and neck was normal, with absent tonsils. Significant findings on DISE included PVCM and a >75% structural obstruction of adenoid hypertrophy.  Table 1 shows a complete listing of the DISE findings.  Video 1 displays the DISE examination. At that time, a revision adenoidectomy was also performed. The results were discussed with her parents with a treatment plan of 10 milligrams of omeprazole with close follow-up visits to monitor the PVCM. At a 4-week follow-up visit, the patient's parents reported that the noisy breathing at night and sleep symptoms had resolved. A repeat polysomnogram eight months after initiating the omeprazole showed an AHI of 0.2 (central apnea only) with an oxygen saturation nadir of 94%. She continues to be asymptomatic with regard to sleep symptoms at 1 year and 4 months after initiation of treatment.

### 2.2. Case 2

A 5-year-old male was noted to have sleep-disordered breathing symptoms of loud snoring, restless sleeping, frequent nocturnal arousals, difficulty in falling asleep, difficulty in awakening in the morning, and reported hyperactive behavior. Daytime breathing was normal. These symptoms warranted a polysomnogram showing an AHI of 1.1 with an oxygen saturation nadir of 94%. Despite a minimally elevated AHI, the patient continued to have significant obstructive symptoms. Head and neck examination was normal with small tonsils. The documented adenoid hypertrophy was noted from a prior nasal endoscopy in conjunction with bilateral ventilation tube removal and septal cauterization for recurrent epistaxis performed by the same pediatric otolaryngologist. The significant results of the DISE revealed >75% adenoid obstruction and a dynamic obstruction of PVCM with high pitched stridor, see Table 1.  Video 2 displays the DISE examination. Adenoidectomy was performed. The patient was started on ranitidine. One month later at the first postoperative visit, his parents indicated that the patient's sleep symptoms were improved.

### 2.3. Case 3

A 6-month-old female was reported to have slept poorly since birth. Parents described nocturnal stridor. She previously was briefly treated with ranitidine, but symptoms persisted. Omeprazole was also prescribed to the patient; however, the patient was not given the medication daily as prescribed. She had a polysomnogram prior to arrival to the otolaryngology clinic showing an obstructive AHI of 17 and a central AHI of 17 with an oxygen saturation nadir of 86%. Head and neck examination was normal with small tonsils. In-office awake laryngoscopic evaluation for nocturnal stridor revealed a normal larynx and no stridor. For these symptoms, she was taken to the operating room with otolaryngology and pediatric pulmonology for DISE where it was noted that she had PVCM and no other site of dynamic obstruction. See Table 1 for a complete listing of the DISE findings.  Video 3 shows the DISE examination. She was started on omeprazole and improved. Her PCP told the mom to stop the medication, and the nocturnal stridor recurred. The mom restarted omeprazole, and the nocturnal stridor disappeared. MRI of the brain and brainstem was normal. Repeat laryngoscopy at 3 months revealed normal vocal cord motion, and her mother reported resolution of her nocturnal symptoms.

## 3. Discussion

OSA affects 2–4% of children in the United States and is associated with numerous negative health sequelae, such as failure to thrive, behavioral deficits, and sudden infant death [2]. OSA symptoms include frequent snoring, presence of gasping or pausing in breathing, daytime sleepiness, difficulty in arousing the child, difficulty in getting the child to sleep, mood swings, irritability, and hyperactivity [3].

Adenotonsillar hypertrophy most commonly and significantly contributes to nocturnal airway obstruction in children [4, 5]. In infants, gastroesophageal reflux disease (GERD) has been noted to trigger OSA via laryngeal chemoreflex [3]. Congenital or acquired upper airway abnormalities such as laryngomalacia, choanal atresia, cleft palate, and subglottic stenosis may also be associated with OSA in infants.

Overnight PSG confirms an OSA diagnosis but does not provide information about the location of the obstruction [3–5]. DISE allows direct visualization of the upper airway to evaluate the site of obstruction. It has been proven to be safe and have good test-retest reliability [6]. There are no contraindications to use of DISE in the pediatric population provided the patient can undergo anesthesia. Indications for the procedure include but are not limited to persistent OSA after adenotonsillectomy such as in Case 1 or significant symptoms of OSA with small tonsils and adenoid as in Case 3 [4]. DISE detected PVCM in all 3 cases but does not contribute to determining the etiology.

PVCM as a contributor to OSA outside of the context of multiple system atrophy (MSA) in adults [7] or other CNS disorders has not been well documented. A case report described rhythmic sounds arising from the vocal cords during sleep did not describe a patient with clinical signs of OSA, and who had an AHI of 0.8 [8]. An additional case report noted the development of PVCM leading to airway obstruction during sleep due to cross-nerve excitement from a vagal nerve stimulator (VNS) [9]. The OSA resolved with decrease in stimulation thresholds from the VNS [9]. Our case series illustrates PVCM as a finding during sleep in children with polysomnogram proven OSA. These children ranged from 6 months to 9 years of age and had no significant comorbidities. Sakthivel et al. previously described a related case, but in a single asthmatic adolescent with a tracheostomy tube in situ [10]. OSA and PVCM are both multifactorial and require etiology-directed treatment [9].

PVCM is characterized by paroxysmal periods of true vocal cord adduction, particularly during inspiration [10]. Normal cord motion will be seen temporally separately as it is an episodic phenomenon. Kuna et al. showed that during hypocapnic apneic pauses in adults, the glottic aperture was smaller than at any other time in the respiratory cycle, and this was associated with electrical activity in the laryngeal adductor muscles [11]. This event may result in the appearance of PVCM. In our case series it is possible that Cases 1 and 2 have PVCM secondary to OSA, but we suspect that Case 3's PVCM was the cause of OSA. We know that none of the children had bilateral vocal cord paresis since none had stridor while awake, and Case 3 had flexible laryngoscopy done while awake showing normal vocal cord motion.

There is no consensus regarding the incidence of PVCM in infants in the literature. The etiological basis for PVCM has been discussed for awake, active patients. Typical exertional or psychiatric causes of PVCM were not likely in these cases, as patients only experienced symptoms at night and were young [12, 13]. Thus, classic PVCM management consisting of psychotherapy, behavioral therapy, or cognitive therapy was not indicated [14]. PVCM was recently presented as the primary symptom of a genetic mutation linked to a rare neuromuscular disorder, paramyotonia congenita; this is also an unlikely cause in our patients given the lack of other neurodegenerative symptoms [15]. Irritant-associated PVCM occurs due to the reflexive closure of the glottic airway after exposure to an irritant [13]. GERD has been linked to PVCM in many cases and is subject to simple treatment with proton pump inhibitors or second-generation histamine antagonists [13, 16]. PVCM in infants has been established in the literature, but the etiology has not been determined [13]. All 3 patients in this series were treated with antireflux medications as part of their therapy, because of the previously reported apparent success of this in infants with PVCM [14]. For the first 2 cases, it is not clear that reflux control played a role in resolution of OSA or if the adenoidectomy was more important. If the PVCM was a sequela of the OSA, the latter would be more likely. All three of the patients have been seen for follow-up and have exhibited improvement or complete resolution of symptoms.

### 3.1. Limitations

Our case series does not determine etiology of PVCM or whether PVCM is a source of the sleep-related airway obstruction in these children. We are simply reporting the finding of PVCM during DISE in the presence of OSA. We cannot determine what interventions contributed to the resolution of OSA. Cases 1 and 2 had adenoidectomies. All three cases have antireflux medications started postoperatively. Collectively, the treatments appear to have resolved the OSA symptoms. Further work will need to be performed to assess the role PVCM plays in pediatric OSA and its management.

## 4. Conclusion

This case series of children with documented OSA had paradoxical vocal cord movement demonstrated with DISE, which suggests that this entity occurs during sleep and may play a role in the etiology of OSA. The vocal cords should be examined closely during DISE.

## Figures and Tables

**Table 1:** DISE findings for each case.

	Case 1	Case 2	Case 3
Nasal airway			
Bilateral nasal airway	Airway narrowed <2 mm at anterior inferior turbinate	Airway narrowed <2 mm at anterior inferior turbinate	Airway narrowed <2 mm at anterior inferior turbinate
Septum	Midline	Midline	Midline

Retropalatal airway			
Adenoid	>75% choanae obstructed	>75% choanae obstructed	<25% choanae obstructed
Soft palate	No movement in respiratory cycle or obstruction	Partially closes airway in anterior-posterior direction	No movement in respiratory cycle or obstruction
Tonsils/lateral pharyngeal walls	Absent, lateral pharyngeal walls medialize during respiration	<25% collapse, no obstruction of airway	<25% collapse without obstruction of airway

Retropalatal airway			
Retrolingual airway patency	Patent	Narrowed, yet patent	Patent
Tongue base	Well positioned, wide open retro-lingual airway, no movement during respiration	Pushes epiglottis posteriorly	Patent vallecula
Epiglottis	Vertical; true vocal cords easily visualized	2-point contact on posterior pharyngeal wall, airway is narrowed, yet patent	Vertical
Lingual tonsil	Normal size	Partially fills vallecula	Normal size

Endolarynx			
Arytenoid and postarytenoid mucosa	No edema, well positioned, no collapse	Vertical without collapse	Vertical without collapse
Collapse of laryngeal mucosa obstructing endolarynx	No obstruction	No obstruction	No obstruction
Vocal cords	Bilateral paradoxical vocal cord motion noted	Bilateral paradoxical vocal cord motion noted	Bilateral paradoxical vocal cord motion noted
Aryepiglottic folds	True and false vocal cords easily visualized	True and false vocal cords easily visualized	True and false vocal cords easily visualized

## Supplementary Materials

Supplementary Materials DISE Examination 1: video recording of Case 1 DISE. Significant findings were dynamic obstruction of PVCM and >75% structural obstruction of adenoid hypertrophy. DISE Examination 2: video recording of Case 2 DISE. Significant findings were >75% structural obstruction of adenoid hypertrophy and a dynamic obstruction of PVCM with high pitched stridor. DISE Examination 3: video recording of Case 3 DISE. The only significant finding was dynamic obstruction of PVCM.
